# Elevated Polyamines in Saliva of Pancreatic Cancer

**DOI:** 10.3390/cancers10020043

**Published:** 2018-02-05

**Authors:** Yasutsugu Asai, Takao Itoi, Masahiro Sugimoto, Atsushi Sofuni, Takayoshi Tsuchiya, Reina Tanaka, Ryosuke Tonozuka, Mitsuyoshi Honjo, Shuntaro Mukai, Mitsuru Fujita, Kenjiro Yamamoto, Yukitoshi Matsunami, Takashi Kurosawa, Yuichi Nagakawa, Miku Kaneko, Sana Ota, Shigeyuki Kawachi, Motohide Shimazu, Tomoyoshi Soga, Masaru Tomita, Makoto Sunamura

**Affiliations:** 1 Division of Gastroenterology and Hepatology, Tokyo Medical University, Shinjuku, Tokyo 160-0023, Japan; yacchan85@yahoo.co.jp (Y.A.); itoi@tokyo-med.ac.jp (T.I.); a-sofuni@amy.hi-ho.ne.jp (A.S.); tsuchiya623@mac.com (T.T.); onakasuicyatta@yahoo.co.jp (Re.T.); tonozuka1978@gmail.com (Ry.T.); 3244honjo@gmail.com (M.H.); maezora1031@yahoo.co.jp (S.M); fujita.com.com@gmail.com (M.F.); kenjirojiro5544@yahoo.co.jp (K.Y.); yukitoshimatsunami1228@yahoo.co.jp (Y.M.); takasikurosawa716@yahoo.co.jp (T.K.); 2 Research and Development Center for Minimally Invasive Therapies Health Promotion and Preemptive Medicine, Tokyo Medical University, Shinjuku, Tokyo 160-8402, Japan; 3 Institute for Advanced Biosciences, Keio University, Tsuruoka, Yamagata 997-0052, Japan; KKK-miku@ttck.keio.ac.jp (M.K.); sana.ota@ttck.keio.ac.jp (S.O.); soga@sfc.keio.ac.jp (T.S.); mt@sfc.keio.ac.jp (M.T.); 4 Third Department of Surgery, Tokyo Medical University, Shinjuku, Tokyo 160-0023, Japan; naga@tokyo-med.ac.jp; 5 Fourth Department of Surgery, Tokyo Medical University Hachioji Medical Center, Hachioji, Tokyo 190-0998, Japan; skawachi@tokyo-med.ac.jp (S.K.); shimazu2401@yahoo.co.jp (Mo.S.); be7@xui.biglobe.ne.jp (Ma.S.)

**Keywords:** pancreatic cancer, saliva, metabolomics, polyamines

## Abstract

Detection of pancreatic cancer (PC) at a resectable stage is still difficult because of the lack of accurate detection tests. The development of accurate biomarkers in low or non-invasive biofluids is essential to enable frequent tests, which would help increase the opportunity of PC detection in early stages. Polyamines have been reported as possible biomarkers in urine and saliva samples in various cancers. Here, we analyzed salivary metabolites, including polyamines, using capillary electrophoresis-mass spectrometry. Salivary samples were collected from patients with PC (*n* = 39), those with chronic pancreatitis (CP, *n* = 14), and controls (C, *n* = 26). Polyamines, such as spermine, *N*_1_-acetylspermidine, and *N*_1_-acetylspermine, showed a significant difference between patients with PC and those with C, and the combination of four metabolites including *N*_1_-acetylspermidine showed high accuracy in discriminating PC from the other two groups. These data show the potential of saliva as a source for tests screening for PC.

## 1. Introduction

Pancreatic cancer (PC) remains one of the cancers with the worst prognoses, and its five-year survival rate is still under 5% [1]. The high mortality rate of PC is caused by the lack of early specific symptoms [2]. The delay in diagnosis may also increase this rate [2]. Recent diagnostic imaging technologies, such as positron emission tomography (PET)-CT, magnetic resonance imaging (MRI), computed tomography (CT), and endoscopic ultrasonography (EUS), have helped to improve the diagnosis of PC. However, 30% of patients present with a locally advanced tumor, 50% present with metastatic disease, and only 20% are diagnosed as resectable at initial diagnosis [3]. Gemcitabine was approved for the treatment of advanced PC in 2001. Gemcitabine, tegafur-gimeracil-oteracil potassium combination (TS-1), multi-agents chemotherapy regimens, such as a regimen consisting of leucovorin calcium, fluorouracil, irinotecan hydrochloride, and oxaliplatin (FOLFIRINOX), and molecular targeted therapeutic agents have already showed effectiveness in the treatment of advanced PC [4]. However, these therapies only contribute to a slight extension of survival with a slight improvement in advanced PC cases. Surgical resection remains the only potentially curative therapy. Thus, early detection is significantly important for improving the survival rate and prognosis of PC. Tumor markers including serum pancreatic enzymes and carbohydrate antigens, such as carbohydrate antigen 19-9 (CA19-9), carcinoembryonic antigen (CEA), s-pancreas-1 antigen (SPan-1), and duke pancreatic monoclonal antigen type 2 (DUPAN-2), have been used for complementary diagnosis [5,6,7]. However, despite the increase in these markers in advanced stage PC, its sensitivity is limited and false negatives have been found in many cases. These tumor markers are not useful for early diagnosis at the time when surgery is possible. Therefore, it is important to establish a simple and inexpensive technology with low invasiveness to efficiently detect early-stage PC.

PC is induced by abnormalities of various tumor-suppressor genes, which accelerate polyamine synthesis and consequently affect various primary pathways [8]. Polyamine synthesis relies on the supply of ornithine, which is converted from arginine by arginase (ARG1 (EC 3.5.3.1)). Ornithine is converted to putrescine by ornithine decarboxylase (ODC) (EC 4.1.1.17), followed by the formation of spermidine by spermidine synthase (SRM) and *S*-adenosylmethionine, which is provided by methionine pathways [9]. ODC is negatively regulated by the interaction with antizyme 1 (OAZ1) [10,11]. OAZ1 regulates both polyamine biosynthesis and transport to maintain homeostasis [10,11].

The drastic change in these metabolisms induced by the mutation of oncogenes were reported in various cancers. The enhanced activity of polyamine pathways in colorectal cancer (CRC) is well known, e.g., the first rate limiting enzyme, ODC is negatively regulated by the adenomatous polyposis coli (APC) tumor-suppressor gene in colonic mucosal tissue [6]. The loss of APC function would activate the ODC enzyme, resulting in the activation of polyamine biosynthesis [7]. The loss of function of p53 and SMAD4, frequently observed in pancreatic cancer, increased polyamine metabolism [12,13]. The upregulation of *MYC*, a downstream of *KRAS*, also increases polyamine biosynthesis and import, leading to high concentrations of intercellular polyamines in cancer cells [14]. These metabolites are secreted from the tumor tissue and spread to surrounding tissues and blood vessels [5]. Therefore, the combination of polyamine or metabolites has been used for the development of non- or low-invasive screening, such as blood, urine, and fecal-based tests [8,15,16,17,18,19], to identify patients with CRC or polyps, a precursor of CRC. Elevated concentration of urinary *N*_1_,*N*_12_-acetylspermine of CRC has been consistently observed in various studies [20,21]. However, all reports have claimed that change in a single polyamine, i.e., low specificity as a biomarker, is not sufficient to diagnose CRC. Nowadays, we have various pattern recognition methods and machine learning algorithms, and the use of these methods has the potential to show better accuracy.

The effect of metabolic changes of PC on blood components has been investigated to explore metabolite-based novel biomarkers to detect patients with PC [17,20,21,22,23,24]. Meta-analysis of these blood metabolomics for PC has also been reported [18]. We also recently reported the diagnostic ability of serum metabolomics [19], indicating the spread of metabolomic change from PC cells to metabolites in blood vessels as well as a range of biofluids.

Various omics technologies revealed the PC detection ability of salivary compounds and microbiomes [25,26,27]. We previously observed a change in metabolite concentration in salivary samples collected from PC patients [28]. Among metabolites, polyamines in non-invasively available biofluids have been reported as possible biomarkers of various cancers [29,30,31]. Elevation of urinary polyamines and the positive correlation between their concentrations in PC tissue is well known [32]. Salivary polyamines have shown potential detection ability in breast cancer [33,34]. However, the potential of PC detection using salivary polyamines has not been investigated.

The purpose of this study was to evaluate the potential ability of salivary polyamines to detect PCs. We utilized capillary electrophoresis-mass spectrometry (CE-MS) to quantify these metabolites and access their sensitivity and specificity by comparison of polyamine profiles for PC, those for chronic pancreatitis (CP), and controls (C).

## 2. Results

Patient information including the number of subjects age, and sex is summarized in Table 1. The same number of female and male subjects were included in C, while larger numbers of males compared with those of females were included in both CP and PC with no significant chi-square value. The average age of PC was higher than those of C and CP at significant levels.

**Figure 1:**
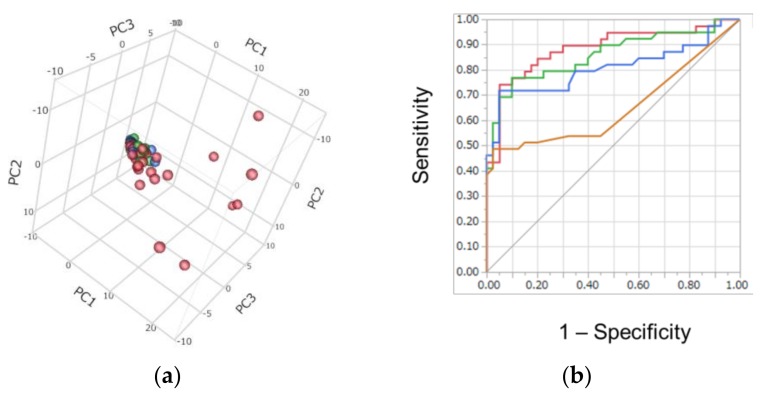
Discrimination ability of metabolomics profile: (**a**) Score plots of principal component analysis (PCA). Contribution ratio to first, second, and third principal component (PC) (PC1, PC2, and PC3) were 34.0, 5.7, and 4.7, respectively. Blue, green, and red plots indicated C, CP, and PC, respectively. (**b**) Receiver operating characteristic (ROC) curves of multiple logistic regression (MLR): red, green, blue, and orange curves indicated the MLR model with 4, 3, 2, and 1 metabolite(s), respectively, and their area under ROC curve (AUC) values were 0.887 (95% confidence interval (CI); 0.784–0.944, *p* < 0.0001), 0.859 (95% CI; 0.749–0.925, *p* < 0.0001), 0.807 (95% CI; 0.749–0.925, *p* < 0.0001), and 0.653 (95% CI; 0.526–0.761, *p* < 0.0122), respectively. The differences in AUC of the model with 4 parameters were 0.0501, 0.0280, and <0.0001 for those with 3, 2, and 1 parameters, respectively.

Metabolomic analysis successfully identified and quantified 292 metabolites in saliva samples. Of these, 142 metabolites were frequently detected (at least >50% per group) and used for subsequent analyses. Score plots of principal component analysis (PCA) (Figure 1a) showed the overall metabolite concentration pattern among all samples. The score plots of C and CP aggregated while several PC plots were scattered, indicating that the metabolomic profiles of PC showed large differences compared to C and CP.

To access the discrimination ability of the salivary metabolites, the receiver operating characteristic (ROC) curve of this multiple logistic regression (MLR) model was developed (Figure 1b). In total, 24 metabolites showed significant differences (corrected *p-*value < 0.05; Mann–Whitney test) and a false change (F.C.) >4.0 between PC and (C + CP), and 4 metabolites were selected via stepwise feature selection (Table 2). The model included alanine, *N*_1_-acetylspermidine, 2-oxobutyrate, and 2-hydroxybutyrate. The *p*-values of all four coefficients were <0.05, so the range of lower and upper 95% confidence interval (CI) of the coefficients did not include 1.0. Among 4 metabolites, only alanine showed a <1.0 odds ratio, indicating that lower concentrations of alanine increase the possibility of PC. Meanwhile, the other three metabolites showed >1.0 odds ratio, indicating that higher concentrations of these metabolites increase the possibility of PC.

The area under the ROC curve (AUC) of this model was 0.887 (95% confidence interval (CI); 0.784–0.944). As pruning, the metabolites showing the largest *p*-value in the model were eliminated one by one. The model with fewer parameters showed lower AUC values.

**Figure 2:**
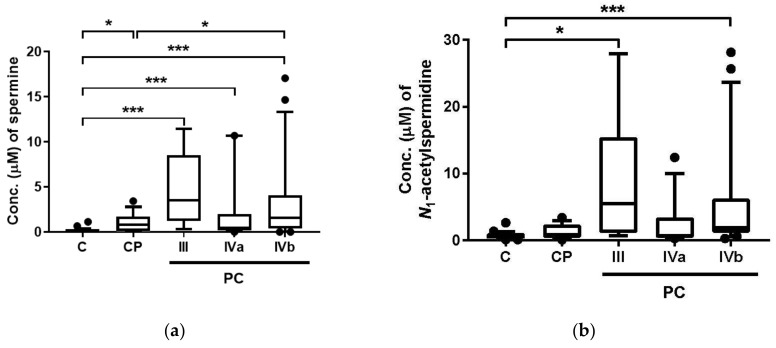

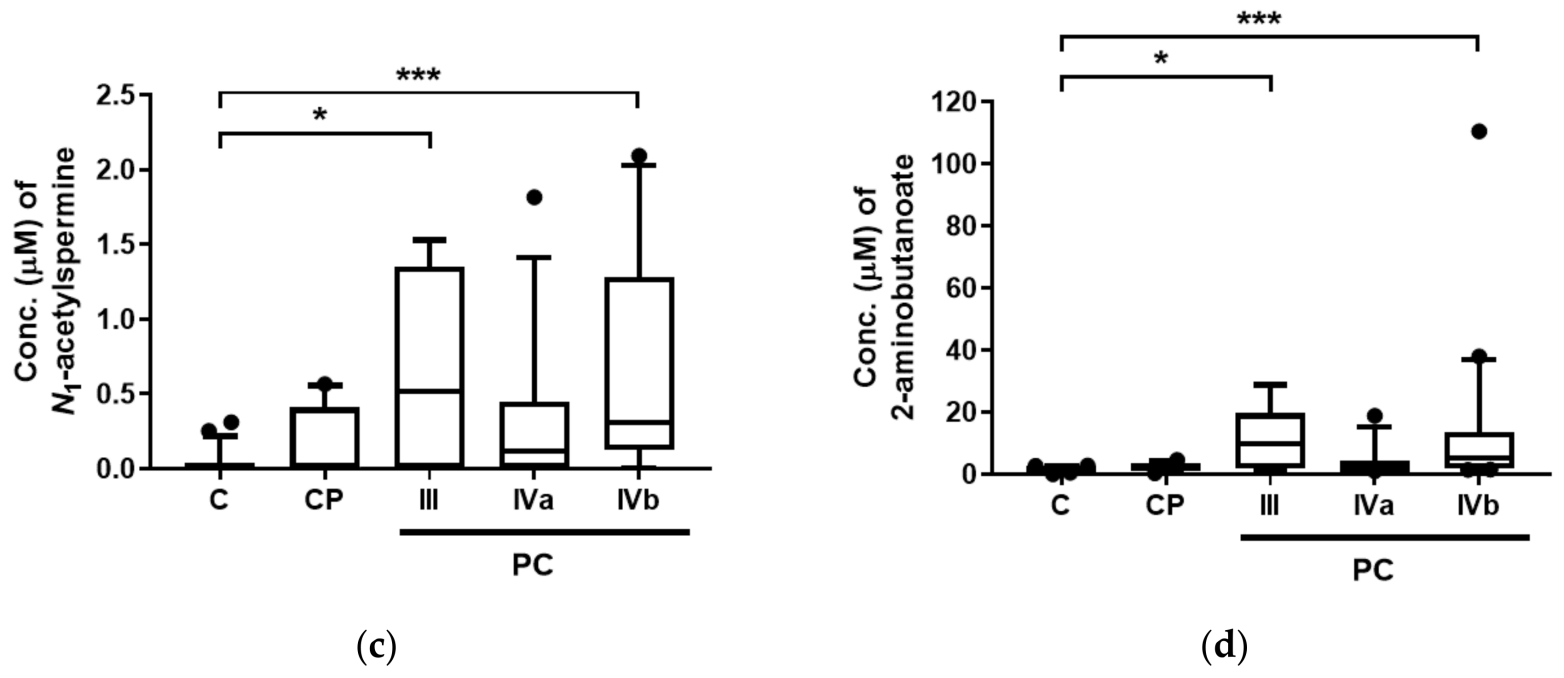
Salivary concentration of four metabolites showing significant difference among control patients (C), chronic pancreatitis patients (CP), and pancreatic cancer patients (PC): (**a**) spermine; (**b**) *N*_1_-acetylspermidine; (**c**) *N*_1_-acetylspermine; (**d**) 2-aminobutanoate (2AB). III, IVa, and IVb indicate the stage of PC. The number of subjects for C, CP, and PC Stages III, IVa, IVb were 26, 14, 6, 12, and 21, respectively. Horizontal bars of box-whisker plots indicate 10%, 90%, median, and lower and upper quantiles. The data <10% and >90% were depicted as plots. *** *p* <0.001 and * *p* < 0.05 by the Steel–Dwass test.

The metabolites showing significant differences between PC and (C + CP) (corrected *p* < 0.001; Mann–Whitney test) included three polyamines (spermine, *N*_1_-acetylspermidine, and *N*_1_-acetylspermine) and 2-aminobutanoate (2AB). Their stage-specific concentrations are depicted in Figure 2. By multiple comparison (Steel–Dwass test), all four metabolites showed significant differences in comparisons between C and PC with Stage III, and C and PC with Stage IVb (Figure 2a–d). In addition, spermine showed significant differences in various comparisons, e.g., comparison between C and PC with Stage IVa, between C and CP, and between CP and PC with Stage IVb (Figure 2a).

Tumor markers, including CEA, CA19-9, DUPAN2, and SPAN1 of CP and PC, are summarized in Table 3. Based on the ROC curve of MLR (Figure 1b), the optimized cut-off value was calculated, and true positive ratios are also summarized in Table 3. The number of positive subjects showing higher value of tumor marker or predicted value by MLR is also summarized. For example, three CP subjects showed false positive (>5.0 ng/mL) for tumor marker CEA. Meanwhile, all subjects with PC Stage III showed false negative. The 75.0% and 47.6% of the subjects with PC Stages IVa and IVb showed true positive for this marker. For all markers and MLR, the averaged values among CP and PC Stages III, IVa, and IVb showed significant difference (*p* < 0.05; Kruskal–Wallis test). Among all tumor markers and MLR, MLR showed the largest *p*-value (*p* = 0.002), indicating a lower difference of averaged values among these groups.

As a computational validation test, *k*-fold cross validation (CV) was conducted using *k* = 5, 10, and 20 with 200 random values for each. The median AUC values were 0.847 (95% CI; 0.840–0.846), 0.850 (95% CI; 0.847–0.851), and 0.852 (95% CI; 0.850–0.853) for *k* = 5, 10, and 20, respectively. The resampling tests were also conducted using 200 random values, which yielded median AUC = 0.894 (95% CI; 0.889–0.901).

## 3. Discussion

We conducted capillary electrophoresis-mass spectrometry (CE-MS)-based metabolomics for comprehensive analyses of hydrophilic metabolites, including polyamines, in saliva samples for individuals from the C, CP, and PC groups. The overall metabolite concentration patterns collected from patients with PC showed large difference among all samples (Figure 1a). Among quantified metabolites, three polyamines, including spermine, *N*_1_-acetylspermidine, and *N*_1_-acetylspermine, and 2-aminobutanoate, showed significant differences between C and PC (Figure 2). The acetylation of polyamines to produce *N*_1_-acetylspermidine and *N*_1_-acetylspermine from spermidine and spermine, respectively, are expected by the activation of spermine/spermidine *N*_1_-acetyltransferase (SSAT) in tumor tissues.

The MLR model developed here included four metabolites and yielded an AUC of 0.887 (Figure 1b). As a pruning test, the AUC values between the model with 4 and 3 metabolites showed no significant difference (*p* = 0.0501, Figure 1b), while the model with 2 and 1 metabolites showed significantly decreased accuracy, indicating that the combination of multiple metabolites contributed to an enhancement of both sensitivity and specificity in discriminating PC patients from the other groups. Alanine was included in this model (Table 2), while no specificity for pancreatic cancer was expected because of the various reports on this metabolite [35,36]. Since not only these 4 metabolites but all 24 metabolites showed potential as markers to discriminate PC from the others (corrected *p* < 0.05; Mann–Whitney test and F.C. > 4.0 between PC and (C + CP)), the specificity of these metabolites should be validated using larger cohorts and parameter selection.

Our computational test using *k*-fold CV and resample tests showed high generalization ability, since no median AUC showed a distinct decrease compared to the original AUC calculated using all datasets. In particular, the differences between the upper margins of the lower limit of the 95% CI of the AUC values of all validation tests were small: 0.006, 0.004, 0.003, and 0.012 for 5, 10, and 20-fold CV and resampling. Both tests indicated a high generalization ability of the model.

Among the metabolites showing high discrimination ability, three polyamines were included (Figure 2). Spermine concentrations showed significant differences in various comparisons; all PC groups at all stages showed significant differences compared with C and PC in Stage IVb (Figure 2a). However, this metabolite also showed a significant difference between C and CP. Two acetylated-polyamines, including *N*_1_-acetylspermidine and *N*_1_-acetylspermine, were elevated in PC compared with Stages III and IVb (Figure 2b,c).

Polyamine catabolism typically involves *N*-acetylation of the aminopropyl end of spermine by spermine/spermidine acetyltransferase (SAT1) to form *N*_1_-acetylspermine and *N*_1_,*N*_12_-diacetylspermine. SAT1 also converts spermidine to *N*_1_-acetylspermidine. The acetylation of these metabolites is also activated in spermidine/spermine *N*_1_-acetyltransferase (SSAT) in cancer cells [8]. In particular, *N*_1_,*N*_12_-diacetylspermine is known to be secreted by tumors and its concentration is elevated in urine in various cancer patients [37]. However, this metabolite was not independently detected in our CE-MS measurement conditions. The problems were caused by the overlap of redundant features, such as adduct or fragment ion of other metabolites. The elevation of salivary *N*_1_-acetylspermidine and *N*_1_-acetylspermine is considered reasonable and the lack of a significant difference between C and PC with Stage IVb might be attributed to the low number of patients.

The combination of salivary metabolites, including polyamines, showed a potential to detect PC subjects (Table 3). The tumor markers were also elevated in PC groups, but the MLR model of salivary metabolites showed the highest number of positive subjects (83.3%) for PC with Stage III, while tumor markers showed lower sensitivities: 0.00%, 50.0%, 66.7%, and 50.0% for CEA, CA19-9, DUPAN2, and SPAN1, respectively. However, CEA is more sensitive (100%) in detecting PC with Stage IVa than MLR (58.3%). Therefore, complementary use of both tumor markers and MLR would reduce the false positive in the PC screening.

There are several limitations that need to be acknowledged. Firstly, the number contained in the cohort of this study was quite small, and validation with a larger cohort is necessary. The difference in age is also a problem of this study. In this study, we recruited only advanced PC in Stages III, IVa, and IVb. The validation of PC at an early stage is most important to access the value of the saliva-based PC screening test demonstrated here. The specificity of the elevated salivary markers should also be analyzed using different types of diseases, since polyamines in saliva samples collected from breast cancer patients were previously reported [33,34]. Our data also revealed a large overlap of elevated salivary metabolites among various cancers [28]. Environmental factors also affect the salivary metabolite profiles [36], and standards of protocols to handle the saliva should also be established based on observed marker metabolites, since several salivary metabolites were unstable after saliva collection [38]. In this study, we followed the protocol of metabolomic analysis [28], which resulted in absolute concentration of each metabolite in saliva samples, and all statistical analyses were conducted using these values. To eliminate the unexpected bias of overall concentration, each metabolite concentration is usually normalized by the creatinine concentration. However, such normalization metabolite has not yet been established. Instead, the use of total protein is one way to normalize the overall concentrations. Exploring the normalization of salivary metabolite is one of issue to be addressed. Taken together, more rigorous validation methods are still necessary to fully evaluate the potential of salivary-based PC detection.

## 4. Materials and Methods

### 4.1. Individual Selection

Sample collection was conducted at Tokyo Medical University Hospital. All patients had pancreatic cancers diagnosed histologically. All patients were recently diagnosed with primary disease and none had received any prior treatment in the form of chemotherapy, radiotherapy, surgery, or alternative therapy. No subjects had a history of prior malignancy. This study was approved by the ethics committee of Tokyo Medical University (approval no. 1560, 30 September 2010). Written informed consent was obtained from all patients and from volunteers who agreed to serve as saliva donors. Our study was carried out in accordance with the Helsinki Declaration.

### 4.2. Protocols for Saliva Collection and Sample Preparation

The saliva providers were not allowed to take any food except water intake after 9:00 p.m. on the previous day. All samples were collected at 8:00–11:00 a.m. The subjects were required to brush their teeth without toothpaste on the day of saliva collection and had to refrain from drinking water, smoking, tooth-brushing, and intense exercise 1 h before saliva collection. They were required to gargle with water just before saliva collection. Approximately 400 μL of unstimulated saliva was collected in a 50 cc polypropylene tube. A polypropylene straw 1.1 cm in diameter was used to assist the saliva collection. After collection, saliva samples were immediately stored at −80 °C until metabolite measurements. The protocol of salivary preparation for metabolomic analyses is described elsewhere [39].

### 4.3. Measurement Conditions and Processing of Raw Data

The metabolite standards, capillary electrophoresis time-of-flight mass spectrometry (CE-TOFMS) instrumentation, and measurement conditions for cationic and anionic metabolites have been previously described [28,39,40,41]. Briefly, CE-TOFMS analysis was performed using an Agilent 7100 CE system (Agilent Technologies, Waldbronn, Germany), an Agilent 6224 liquid chromatography (LC)/MS TOF system, an Agilent 1260 series isocratic HPLC pump, a G1603A Agilent CE-MS adapter kit, and a G1607A Agilent CE-ESI-MS sprayer kit (Agilent Technologies, Santa Clara, CA, USA). For system control and data acquisition, Agilent Chemstation software was used for CE, and Agilent MassHunter software (B.02.01.SR1, Agilent Technologies, Santa Clara, CA, USA) was used for TOF-MS data analyses.

Analysis of raw data was conducted by following the typical data processing flow [35]. The flow included filtering noise, subtraction of baselines, integration of peaks of each sliced electropherogram, estimation of accurate *m*/*z* in a mass spectrometry, alignment of multiple datasets to generate peak matrix, and identification of each peak by matching the *m*/*z* value and the corrected migration time to corresponding entries in a standard library, using MasterHands (Keio University, Tsuruoka, Japan) [28]. Metabolite concentrations were calculated based on a ratio of peak area divided by the area of the internal standards between sample and standard compound mixtures.

### 4.4. Statistical Analysis

The Mann–Whitney test was used to access the difference of metabolite concentrations between 2 groups. The false discovery rate (Benjamini and Hochberg methods) [42] was used to correct *p*-values, considering multiple independent tests. Clinical values, except for continuous values, were accessed by the χ^2^ test. Overall metabolomic concentrations were accessed by principal component analysis (PCA). To eliminate noise-like peaks, only frequently detected metabolites (50% of subjects of at least one group) were used for PCA. To evaluate the discrimination ability of multiple metabolites, multiple logistic regression (MLR) was conducted. Of the metabolites used for PCA, metabolites showing both significant differences (corrected *p*-value <0.05 by Mann–Whitney test) and fold change (F.C.) >4.0 of the averaged concentrations between PC and (C + CP) groups were selected. Subsequently, stepwise feature selection with backward (*p* > 0.05) and forward selection (*p* < 0.05) to eliminate multicollinearity and an MLR model was developed. The Steel–Dwass test was used for stage-specific differences.

To access the generalization ability of MLR, two computational validations were conducted: (1) *k*-fold cross validation (CV) and (2) resampling. For the former, data were randomly split into two (*k*: *k*−1) datasets and the former was used for training, the remaining data were used for validation. This was repeated *k* times and the prediction ability using validation datasets was used. For the latter, to eliminate optimistic prediction, subjects were randomly selected, allowing redundant selection to generate the datasets with the number of subjects identical to the original datasets, the MLR model was developed and the accuracy was accessed.

The analyses were conducted using R (ver. 3.4.3, R Foundation for Statistical Computing, Vienna, Austria) [43], JMP (ver. 13.2.0, SAS Institute Inc., Cary, NC, USA), and WEKA (ver. 3.6.13) [44], and GraphPad Prism (ver 7.03, GraphPad Software Inc., San Diego, CA, USA).

## 5. Conclusions

In this study, we evaluated salivary metabolite concentration patterns among PC, CP, and C. Polyamines, especially spermine, showed a significant difference between C and PC. The combination of four metabolites showed high accuracy in discriminating PC from CP and C, and computational validation tests confirmed the high generalization ability of the developed model. There are several limitations, and we should further validate our findings with larger cohorts, including both early-stage PC and other cancer patients; however, the salivary metabolites including polyamines show potential for use in tests screening for PC.

## Figures and Tables

**Table 1:** Characteristics of the subjects involved in this study.

Parameters	C	CP	PC	*p*-value	
n	26	14	39	-	
Age ^1^	50.8 ± 16.4	51.1 ± 12.4	66.1 ± 9.86	<0.0001	***
Sex (F/M) ^2^	13/13	3/11	18/21	0.189	

Note: ^1^ The values indicate average ± standard deviation. *p*-value was calculated by Mann–Whitney test. ^2^ The values indicate the number of female and male subjects. *p*-value was calculated by the χ^2^ test. C: control CP: chronic pancreatitis; PC: pancreatic cancer; n: number of subjects; ***: *p* < 0.001.

**Table 2:** Parameters of the multiple logistic regression (MLR) model.

Markers	Unit Odds Ratio ^1^	Coefficients ^1,2^	*p*-Value
alanine	0.990 (0.980–1.00)	−0.0103 (−0.0203–−0.0003)	0.043 *
*N*_1_-acetylspermidine	2.92 (1.35–6.31)	1.07 (0.30–1.84)	0.0065 ***
2-oxobutyrate	1.15 (1.02–1.29)	0.14 (0.02–0.25)	0.019 *
2-hydroxybutyrate	1.46 (1.07–1.99)	0.38 (0.07–0.69)	0.017 *
(Intercept)	-	−2.21 (−3.21–−1.21)	<0.0001 ***

Note: ^1^ Values were depicted with lower and upper 95% confidential intervals. ^2^ Coefficients indicate the estimated coefficients in the multiple logistic regression model. *: *p* < 0.05 ***: *p* < 0.001.

**Table 3:** The number of positive subjects of tumor markers and the MLR model.

Marker ^1^	CP ^2^ (*n* = 14)	PC ^3^			*p*-Value ^4^
		III (*n* = 6)	IVa (*n* = 12)	IVb (*n* = 21)	
CEA	3.46 ± 3.27	2.75 ± 1.46	7.25 ± 4.18	43.8 ± 101	0.0196 *
>5.0 ng/mL	3 (21.4)	0 (0.00)	9 (75.0)	10 (47.6)	
#MV ^5^	0	0	0	0	
CA19-9	19.9 ± 21.3	2.90 × 10^2^ ± 6.26 × 10^2^	7.43 × 10^2^ ± 9.98 × 10^2^	6.25 × 10^3^ ± 1.77 × 10^4^	0.0016 ***
>37 U/mL	2 (14.3)	3 (50.0)	12 (100.0)	16 (76.2)	
#MV ^5^	0	0	0	0	
DUPAN2	74.0 ± 86.7	6.17 × 10^2^ ± 7.67 × 10^2^	5.28 × 10^2^ ± 6.52 × 10^2^	9.98 × 10^2^ ± 6.52 × 10^2^	0.0008 ***
>150 U/mL	2 (16.7)	4 (66.7)	6 (50.0)	18 (90.0)	
#MV ^5^	2	0	0	1	
SPAN1	16.2 ± 17.1	89.0 ± 1.58 × 10^2^	3.63 × 10^2^ ± 4.83 × 10^2^	3.25 × 10^3^ ± 8.44 × 10^3^	<0.0001 ***
>30 U/mL	2 (18.2)	3 (50.0)	10 (83.3)	19 (95.0)	
#MV ^5^	3	0	0	1	
MLR	0.334 ± 0.266	0.800 ± 0.299	0.633 ± 0.363	0.786 ± 0.268	0.002 ***
> 0.5533	2 (14.3)	5 (83.3)	7 (58.3)	16 (76.2)	
#MV ^5^	0	0	0	0	

Note: Values for markers and MLR were mean ± standard deviations. ^1^ Marker indicates tumor marker or multiple logistic regression (MLR) with their thresholds. The (%) indicates the percentage of positive subjects. ^2^ CP and ^3^ PC indicate chronic pancreatitis patients and pancreatic cancer patients, respectively. ^4^ Kruskal–Wallis test. ^5^ #MV indicates the number of missing values (MV). CEA: carcinoembryonic antigen; CA19-9: carbohydrate antigen 19-9; CEA: carcinoembryonic antigen; Span-1: s-pancreas-1 antigen; DUPAN-2: duke pancreatic monoclonal antigen type 2, *: *p* <0.05 ***: *p* < 0.001.
